# Impact of ovariectomy, high fat diet, and lifestyle modifications on oxidative/antioxidative status in the rat liver

**DOI:** 10.3325/cmj.2014.55.218

**Published:** 2014-06

**Authors:** Rosemary Vuković, Senka Blažetić, Ivana Oršolić, Marija Heffer, Sandor G. Vari, Martin Gajdoš, Zora Krivošíková, Patrícia Kramárová, Anton Kebis, Elizabeta Has-Schön

**Affiliations:** 1 Department of Biology, J. J. Strossmayer University of Osijek, Osijek, Croatia; 2 Department of Medical Biology, School of Medicine, J. J. Strossmayer University of Osijek, Osijek, Croatia; 3 International Research and Innovation Management Program, Cedars-Sinai Medical Center, Los Angeles, CA, USA; 4 Faculty of Medicine, Slovak Medical University in Bratislava, Bratislava, Slovakia; 5 Faculty of Public Health, Slovak Medical University in Bratislava, Bratislava, Slovakia

## Abstract

**Aim:**

To estimate the impact of high fat diet and estrogen deficiency on the oxidative and antioxidative status in the liver of the ovariectomized rats, as well as the ameliorating effect of physical activity or consumption of functional food containing bioactive compounds with antioxidative properties on oxidative damage in the rat liver.

**Methods:**

The study was conducted from November 2012 to April 2013. Liver oxidative damage was determined by lipid peroxidation levels expressed in terms of thiobarbituric acid reactive substances (TBARS), while liver antioxidative status was determined by catalase (CAT), glutathione peroxidase (GPx), glutathione S-transferase (GST), glutathione reductase (GR) activities, and glutathione (GSH) content. Sixty-four female Wistar rats were divided into eight groups: sham operated and ovariectomized rats that received either standard diet, high fat diet, or high fat diet supplemented with cereal selenized onion biscuits or high fat diet together with introduction of physical exercise of animals.

**Results:**

High fat diet significantly increased TBARS content in the liver compared to standard diet (*P* = 0.032, *P* = 0.030). Furthermore, high fat diet decreased the activities of CAT, GR, and GST, as well as the content of GSH (*P* < 0.050). GPx activity remained unchanged in all groups. Physical activity and consumption of cereal selenized onion biscuits showed protective effect through increased GR activity in sham operated rats (*P* = 0.026, *P* = 0.009), while in ovariectomized group CAT activity was increased (*P* = 0.018) in rats that received cereal selenized onion biscuits.

**Conclusion:**

Feeding rats with high fat diet was accompanied by decreased antioxidative enzyme activities and increased lipid peroxidation. Bioactive compounds of cereal selenized onion biscuits showed potential to attenuate the adverse impact of high fat diet on antioxidative status.

Reactive oxygen species (ROS) are common by-products of many oxidative biochemical and physiological processes, and are also involved in numerous physiological and pathophysiological processes. While in low concentrations they may be beneficial in processes such as intracellular signaling and defense against microorganisms, higher concentrations cause cell damage via oxidative modification of proteins, lipids, and DNA, and thus play a major role in the pathogenesis of a variety of human diseases (1). The balance between production and neutralization of ROS is maintained by antioxidant defense system. The system includes antioxidant enzymes such as superoxide dismutase (SOD), catalase (CAT), glutathione peroxidase (GPx), glutathione reductase (GR), and glutathione S-transferase (GST), and a number of low mass non-enzymatic molecules that are scavenging ROS, such as glutathione (GSH) (2,3). An imbalance between ROS production and the cellular antioxidant defense system leads to oxidative stress, which results in lipid peroxidation (LPO) and increased tissue injury (4,5). In liver tissue, this process leads to fibrosis, chronic inflammation, and apoptosis (6).

It has been postulated that oxidative processes and antioxidant defense can be sex-related (7). Such sex-related differences may be due to gonadotropic hormones, primarily estrogens (8). Estradiol and its derivatives are strong endogenous antioxidants that reduce LPO levels in the liver and serum (9,10). Also, estrogens can up-regulate the expression of antioxidative enzymes, such as GPx and SOD (10-12). There is evidence that imbalance in oxidative and antioxidative status is present in women during postmenopausal life (13). The lack of protective action of estrogens is known to cause serious metabolic disturbances, and oxidative stress is thought to be one of the suspected mechanisms (14). Ovariectomy in rats is a commonly used animal model for elucidating the impact of estrogen insufficiency and metabolic consequences (15,16). Estrogen insufficiency is often associated with increased food intake and body weight, therefore high fat diet (HFD)-induced obesity could be an additional problem in menopausal women, and it could affect the levels of oxidative stress in the liver.

Feeding rats with HFD was proved to be a useful model of effects of dietary fat in humans (17). HFD is considered as a major risk factor for a numerous diseases, including metabolic disorders and cardiovascular diseases (CVD). Feeding a HFD for a long time results in the occurrence of nonalcoholic fatty liver disease (NAFLD) (18). Recent studies have suggested that a fundamental role in development of these disorders is played by oxidative stress (19). Oxidative stress, being one of the key pathophysiological mechanisms in liver disease associated with obesity, may also serve as a predictor of CVD (18,20). Due to its significant role in disease development, increased oxidative stress remains a potential attractive target for prevention and therapy of adverse HFD and ovariectomy effects. The impact of HFD and estrogen deficiency on oxidative stress can be reduced by regular physical activity (21,22) and intake of phytochemical-rich foods or supplements (19,23). Recently, numerous in vitro and animal studies have provided evidence that polyphenols may be protective against oxidative-triggered pathologies (24,25).

The aim of this study was to estimate the effect of HFD on the oxidative and antioxidative status in the liver of ovariectomized (OVX) rats, and to investigate the possible ameliorating effect of lifestyle modifications, such as physical activity or consuming functional foods – cereal selenized onion biscuits (SOB) with bioactive complex – on oxidative damage in the liver.

## Materials and methods

### Animals and study design

All experiments were conducted in accordance with the current legislation on the use of experimental animals in Slovakia and with the approval of the Ethics Committee for Animal Experiments of the Slovak Medical University and of the State Veterinary and Food Authority of the Slovak Republic. All experimental procedures were carried out in animal facility in compliance with the standard operating procedures of the Department of Toxicology of the Slovak Medical University and the European Convention for the Protection of Vertebrate Animals used for Experimental and other Scientific Purposes. The study was conducted from November 2012 to April 2013.

**Figure 1:**
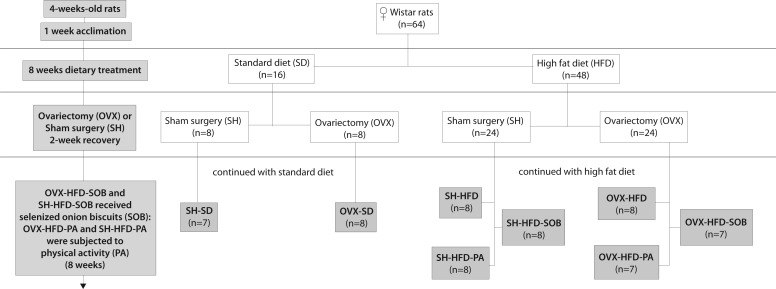
Study flow diagram. Gray squares represent the final eight groups of the animals. The final number of rats per group was 7-8, because some animals were excluded from the study, due to abdominal infections and development of axillary tumor.

Sixty-four female Wistar rats were obtained from Charles River Wiga GmbH (Sulzfeld, Germany). Rats were ~ 4 weeks old, weighed 130-150 g, and were housed in an air-conditioned room (humidity 55 ± 5%, temperature 22 ± 2°C) under a 12:12 hours light/dark cycle with *ad libitum* food and water access. After one-week acclimation period, the rats were randomly divided into two dietary groups: 16 rats received a standard diet (StD; M3, Bonagros.r.o., Blazovice, Czech Republic), while 48 rats received a HFD (D12451 /I/ mod. 45 kJ% fat, ssniff Spezialdiätten GmbH, Soest, Germany). Following the eight-week dietary intervention, rats from both dietary groups were subjected to either ovariectomy (OVX rats) or sham surgery (SH rats) (Figure 1). Bilateral ovariectomy was performed on 32 rats (8 from StD group, 24 from HFD group) using a single dorso-lateral approach (26,27), while the remaining 32 rats from both dietary groups (8 from StD group, 24 from HFD group) were subjected to sham surgery. After the two week recovery period, all animals continued with StD or HFD for the following 8 weeks. In addition, HFD group of animals was further randomly divided into three equal groups as follows: the first sub-group of rats (8 OVX and 8 SH) continued to receive HFD only, the second (8 OVX and 8 SH) received food supplements to their HFD in the form of cereal SOB. The SOBs contained bioactive compounds such as selenium in organic form, quercetin, curcumin, and catechins (28). The third sub-group of rats (8 OVX and 8 SH) was additionally subjected to physical activity (PA rats) in the form of exercise on a 4-channel treadmill (Harvard Apparatus, Holliston, MA, USA). The exercise consisted of a 2-week accommodation phase with increasing exercise intensity (first week: 15-18 m/min for 10-30 minutes, second week: 18-20 m/min for 30-60 minutes), followed by an eight-week constant training period (20 m/min for 60 minutes). Before each training session (5 times a week, always between 8.00 and 9.00 am), all running animals had a 5-minute warm-up phase with a slowly increasing speed. Animals from sedentary groups were placed for the same period on a turned-off treadmill. Due to abdominal infections and development of axillary tumor, some animals were excluded from the study; therefore the number of animals in some groups was seven. The final groups of animals were as follows:

Sham operated rats:

1) SH-StD – rats fed a StD (n = 7)

2) SH-HFD – rats fed a HFD (n = 8)

3) SH-HFD-PA – rats fed a HFD and subjected to PA (n = 8)

4) SH-HFD-SOB – rats fed a HFD supplemented with SOB (n = 8)

Ovariectomized rats:

5) OVX-StD – rats fed a StD (n = 8)

6) OVX-HFD – rats fed a HFD (n = 8)

7) OVX-HFD-PA – rats fed a HFD and subjected to PA (n = 7)

8) OVX-HFD-SOB – rats fed a HFD supplemented with SOB (n = 7)

### Sample collection

By the end of the experimental period, all rats were sacrificed, and the livers were collected by manual dissection, washed twice with ice-cold saline, and blotted on filter-paper. Immediately after, tissue samples were flash-frozen in liquid nitrogen and stored at -80°C until analysis.

### Preparation of tissue extracts

Frozen tissue samples were grounded in a pestle and mortar with liquid nitrogen and the powder was aliquoted into four tubes and weighed. Aliquoted tissue powder was homogenized in an adequate solution using Ultra turrax T10 homogenizer (1300 rpm; IKA, Königswinter, Germany) while kept on ice. For the LPO determination, tissue was homogenized in ice-cold 1.15% KCl solution (1:10, w/v). For determining the GSH levels, tissue was homogenized (1:10, w/v) in 5% 5-sulfosalicylic acid solution (SSA), then maintained on ice for 10-minute and centrifuged at 10 000 g for 10-minute at 4°C. For GPx and GR activity assay, liver tissue was homogenized (1:10, w/v) in 50 mM phosphate buffer (pH 7.8) and for CAT and GST activity assay (1:10, w/v) in 100 mM phosphate buffer (pH 7.0) containing 1 mM EDTA. Crude tissue homogenates were sonicated for 30 seconds while kept on ice in three 10 seconds-intervals, then centrifuged at 20 000 g for 15-minute at 4°C. GSH content and LPO products in the liver homogenates were determined immediately following homogenate preparation, while aliquots of the resulting supernatant for determination of enzyme activities were stored in plastic tubes at -70°C until assayed. The absorbance of LPO product, GSH content, and enzyme activity assay was recorded using a Lambda 2 UV-Vis spectrophotometer equipped with UV WinLab software package (Perkin Elmer, Wiesbaden, Germany).

### Determination of lipid peroxidation

The LPO levels in collected hepatic tissue were estimated by measuring the thiobarbituric acid reactive substances (TBARS), according to the method described by Ohkawa et al (29). This method is based on the formation of red pigment, generated by reaction of LPO breakdown products like malondialdehyde (MDA) with thiobarbituric acid (TBA) at an optimum pH of 3.5. Briefly, tissue homogenate (10%, w/v) was mixed with sodium dodecyl sulfate, acetate buffer (pH 3.5), and an aqueous solution of TBA. After heating at 95°C for 60-minute, the produced red pigment was extracted with n-butanol-pyridine mixture and estimated by the absorbance at 532 nm. The results were expressed as nmol/mg of fresh tissue (FW) according to a standard curve, which was prepared using 1,1,3,3-tetramethoxypropane as a standard.

### Measurement of total glutathione content

Total GSH content in the liver was determined using a kinetic method based on a continuous reduction of 5,5-dithiobis (2-nitrobenzoic acid) (DTNB) to 5-thio-2-nitrobenzoic acid (TNB) by catalytic amounts of reduced glutathione, where the oxidized glutathione form is recycled by GR and NADPH (30). The formation of TNB was continuously recorded at 412 nm at 25°C. Briefly, after deproteinization with the SSA, the resulting supernatant was transferred to the reaction mixture that contained 100 mM phosphate buffer with 1 mM EDTA (pH 7.0), 0.031 mg/mL DTNB, and 0.115 units/mL of GR in a final volume of 1.05 mL. The mixture was incubated at 25°C for 5-minute and the reaction was initiated by adding NADPH at a final concentration of 48 μM. The total amount of GSH was determined by a standard curve of reduced GSH, and the results were expressed as nmol/mg of FW.

### Antioxidant enzyme activities assay

GST (EC 2.5.1.13) activity was determined by measuring the conjugation of 1-chloro-2,4-dinitro benzene (CDNB) with reduced glutathione that produced a dinitrophenylthioether, which was accompanied by an increase in absorbance at 340 nm (31). The assay mixture consisted of 100 mM phosphate buffer with 1 mM EDTA (pH 6.5), 2.5 mM GSH, and 1 mM CDNB, in a final volume of 1.5 mL. One unit conjugates 1.0 μmole of 1-chloro-2,4-dinitrobenzene with reduced glutathione per minute at pH 6.5 and 25°C. GST activity was calculated using molar extinction coefficient of glutathione-1-chloro-2,4-dinitrobenzene conjugate (ϵ = 9.6 mM/cm) and expressed as U/mg protein.

GR (EC 1.6.4.2) was determined by the measurement of the consumption of NADPH during the reduction of GSSG, as demonstrated by a decrease in absorbance at 340 nm. The assay mixture consisted of 1 mM GSSG and 0.1 mM NADPH in 100 mM phosphate buffer containing 1 mM EDTA (pH 7.5). One unit reduces 1.0 μmol of oxidized glutathione per minute at pH 7.5 and 25°C. GR activity was calculated using molar extinction coefficient for NADPH (ϵ = 6.220 mM/cm) and expressed as U/g protein (32).

CAT (EC 1.11.1.6) activity was estimated spectrophotometrically using H_2_O_2_ as a substrate (33). The reaction mixture consisted of 10 mM H_2_O_2_ in 50 mM phosphate buffer pH (7.0). Changes in absorbance in the reaction mixture were measured at 240 nm during 30 seconds after adding the sample. One unit of activity corresponds to the loss of 1 μmol of H_2_O_2_ per minute. CAT activity was calculated using molar extinction coefficient (ϵ = 0.04 mM/cm) and expressed as U/mg protein.

GPx (EC 1.11.1.9) activity was measured according to a modified method described by Wendel (34), using H_2_O_2_ as a substrate. According to this method, GPx activity was determined indirectly by measuring the rate of NADPH oxidation to NADP^+^, accompanied by a decrease in absorbance at 340 nm. The assay mixture consisted of 50 mM phosphate buffer with 0.4 mM EDTA and 1 mM sodium azide (pH 7.0), 0.12 mM NADPH, 3.2 units of GR, 1 mM glutathione, and 0.0007% (w/w) hydrogen peroxide in a total volume of 1.55 mL. One unit catalyzes the oxidation by H_2_O_2_ of 1.0 μmole of reduced glutathione to oxidized glutathione per minute at pH 7.0 and 25°C. GPx activity was calculated using molar extinction coefficient for NADPH (ϵ = 6.220 mM/cm) and expressed as U/mg protein.

### Determination of protein concentration

Total soluble protein concentration in protein extracts was estimated following the protocol described by Bradford (35), using bovine serum albumin as a standard.

### Statistical analyses

The data are presented as mean ± standard deviations (SD) for 7-8 animals in each group and analyzed using STATISTICA 8.0 software package (StatSoft Inc., Tulsa, OK, USA). Differences among groups were assessed by a one way analysis of variance (ANOVA), followed by a *post hoc* analysis using Duncan’s multiple range test. A mean difference was significant at the 0.05 level. Correlation between the analyzed parameters was evaluated using Pearson correlation coefficient with the level of significance <0.05.

## Results

### Effect of HFD, PA, and SOB on TBARS content in the liver of OVX rats

**Figure 2:**
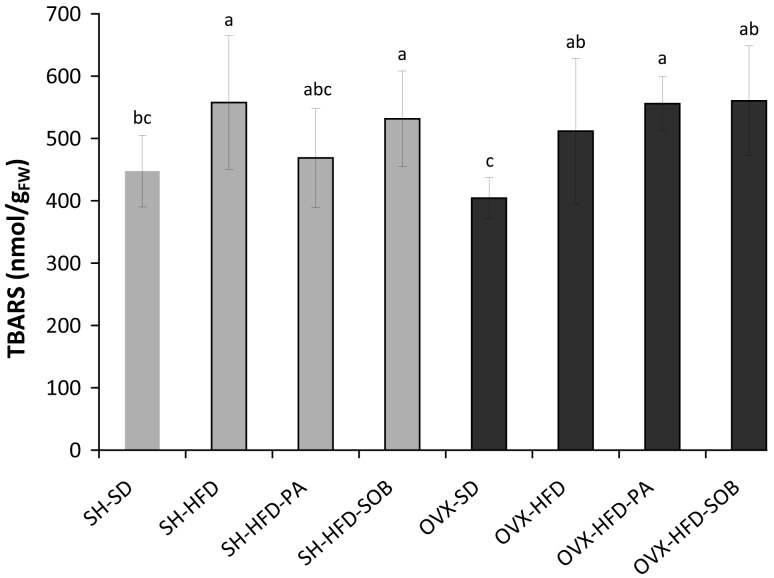
Thiobarbituric acid reactive substances (TBARS) content in the liver of sham-operated (SH) and ovariectomized (OVX) rats fed with standard diet or high fat diet (SH-StD, SH-HFD, OVX-StD, OVX-HFD), SH and OVX rats that received HFD and were subjected to physical activity (SH-HFD-PA, OVX-HFD-PA), and SH and OVX rats that received HFD supplemented with selenized onion biscuits (SH-HFD-SOB, OVX-HFD-SOB). Results are presented as means ± standard deviation. Different letters denote significant differences between the groups (*P* < 0.05), while letters shared in common indicate no significant difference between the groups.

HFD significantly increased TBARS content in both, SH and OVX rats, compared to the control groups that received StD (SH-StD, *P* = 0.032; OVX-StD, *P* = 0.030). The TBARS content in OVX-HFD group was 27% higher and in SH-HFD group was 25% higher than in the corresponding control groups. Neither PA nor SOB supplements induced significant changes in TBARS content in SH and OVX group that received HFD. Ovariectomy did not affect LPO levels; no significant differences were observed in the TBARS content between the OVX and the corresponding SH groups (Figure 2).

### Effect of HFD, PA, and SOB on GSH content in the liver of OVX rats

**Figure 3:**
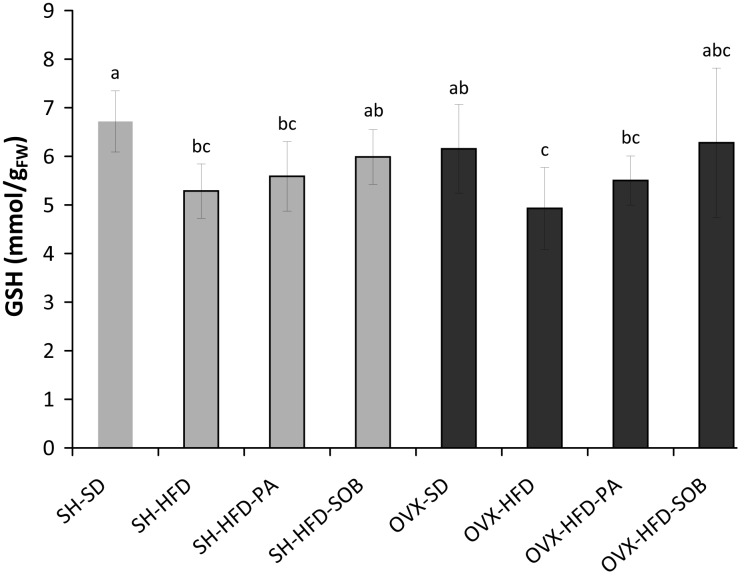
Glutathione (GSH) content in the liver of sham-operated (SH) and ovariectomized (OVX) rats fed with standard diet or high fat diet (SH-StD, SH-HFD, OVX-StD, OVX-HFD), SH and OVX rats that received HFD and were subjected to physical activity (SH-HFD-PA, OVX-HFD-PA), and SH and OVX rats that received HFD supplemented with selenized onion biscuits (SH-HFD-SOB, OVX-HFD-SOB). Results are presented as means ± standard deviation. Different letters denote significant differences between the groups (*P* < 0.05), while letters shared in common indicate no significant difference between the groups.

HFD reduced GSH levels in both SH and OVX rats compared to the groups that received StD (SH-StD, *P* = 0.010; OVX-StD, *P* = 0.026). The GSH content in OVX-HFD group was 27% lower and in SH-HFD group was 29% lower than in the control groups. Neither PA nor SOB supplements induced significant changes in GSH content in SH and OVX group that received HFD. In addition, there were no significant differences in the GSH levels between OVX and the corresponding SH group (Figure 3).

### Effect of HFD, PA, and SOB on GST activity in the liver of OVX rats

**Figure 4:**
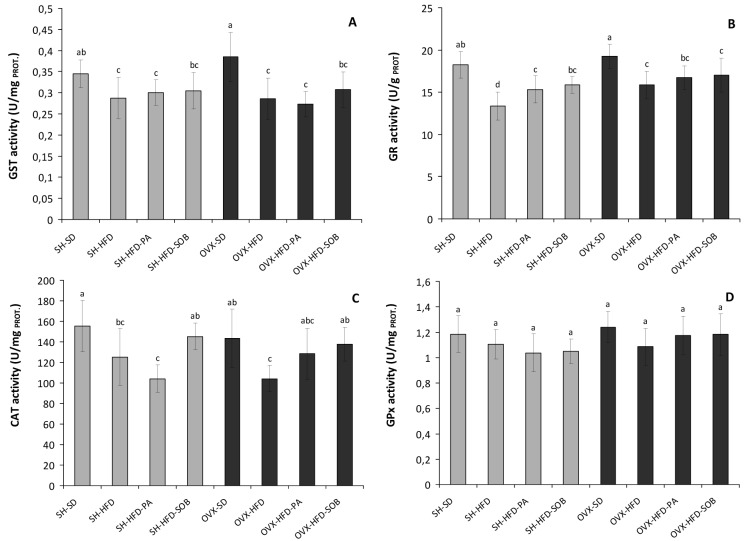
Glutathione S-transferase (GST) (**A**), glutathione reductase (GR) (**B**), catalase (CAT) (**C**), and glutathione peroxidase (GPx) (**D**) activity in the liver of sham-operated (SH) and ovariectomized (OVX) rats fed with standard diet or high-fat diet (SH-StD, SH-HFD, OVX-StD, OVX-HFD), SH and OVX rats that received HFD and were subjected to physical activity (SH-HFD-PA, OVX-HFD-PA), and SH and OVX rats that received HFD supplemented with selenized onion biscuits (SH-HFD-SOB, OVX-HFD-SOB). Results are presented as means ± standard deviation. Different letters denote significant differences between the groups (*P* < 0.05), while letters shared in common between the groups indicate no significant difference.

HFD significantly reduced GST activity in the OVX rats, as well as in SH rats compared to the control groups that received StD (OVX-StD, *P* < 0.001; SH-StD, *P* = 0.035). In SH-HFD group, GST activity was 17% lower and in OVX-HFD group it was 26% lower than in the corresponding control groups. However, no significant differences in GST activity were observed between OVX and the corresponding SH groups. Neither PA nor SOB induced significant changes in GST activity in SH and OVX rats that received HFD (Figure 4A).

### Effect of HFD, PA, and SOB on GR activity in the liver of OVX rats

HFD significantly reduced GR activity in SH-HFD rats for 27% and in OVX-HFD rats for 18%, compared to the control groups that received StD (SH-StD, *P* < 0.001; OVX-StD, *P* < 0.001). No significant difference between the OVX and SH rats that received StD was found, while OVX rats that received HFD showed significantly higher GR activity than SH-HFD group (*P* = 0.008). PA induced significant increase of 15% (*P* = 0.026) in GR activity in SH-HFD-PA rats compared to the SH-HFD group, while no difference in OVX group was found. Also, SOB significantly increased GR activity in SH-HFD-SOB group for 19% compared to the SH-HFD group (*P* = 0.009). On the other hand, supplement biscuits did not affect GR activity in OVX group (Figure 4B).

### Effect of HFD, PA, and SOB on CAT activity in the liver of OVX rats

As was the case with other antioxidative enzymes, HFD reduced the CAT activity in both SH and OVX rats, compared to the control groups that received StD (SH-StD, *P* = 0.040 OVX-StD, *P* = 0.007). In SH-HFD group, CAT activity was 19% lower and in OVX-HFD group it was 27% lower than in the corresponding control groups. No significant changes were observed between OVX rats that received HFD and StD and SH rats that received HFD and StD. SH and OVX rats that received HFD and were additionally subjected to PA did not show any changes in CAT activity when compared to the SH-HFD and OVX-HFD groups. SOB significantly increased CAT activity in OVX-HFD-SOB group (32%, *P* = 0.018) compared to the OVX-HFD group. On the other hand, supplement biscuits did not affect CAT activity in SH rats (Figure 4C).

### Effect of HFD, PA, and SOB on GPx activity in the liver of OVX rats

Although there were no significant differences in GPx activity between all experimental groups (Figure 4D), there was a positive correlation between GPx activity and other antioxidative enzymes (GST, r = 0.407, *P* = 0.001; CAT, r = 0.418, *P* = 0.002; GR, r = 0.407, *P* = 0.001).

## Discussion

### Impact of ovariectomy and HFD on oxidative/antioxidative status in the rat liver

The present study showed higher TBARS levels in OVX and SH animals that received HFD. Elevated TBARS levels in the liver are an evident manifestation of excessive formation of free radicals and activation of LPO. Furthermore, our results revealed a significant decrease in the hepatic GST, GR, and CAT activities, as well as a decrease in hepatic GSH level in OVX and SH rats that received HFD. Therefore, feeding rats with HFD resulted in increased hepatic tissue oxidative stress, which is characterized by reduced antioxidant defense mechanisms and increased LPO in liver tissues of both SH and OVX rats. Although different antioxidative response to HFD of SH and OVX animals was expected, there was no influence of ovariectomy on oxidative status and no interaction effect between HFD and ovariectomy.

It is well known that ovariectomy results in general changes in metabolism, which are detected in the liver (14). The influence of estrogen insufficiency and metabolic disturbances on the liver is important from the clinical point of view because it may play a role in developing liver diseases through the generation of ROS (36,37). The lack of protective action of estrogens is reflected in alterations in antioxidative/oxidative balance in the rat liver (14). Kankofer et al (14) showed an increase in LPO intensity, GPx activity, and total antioxidant capacity in OVX rats, suggesting higher demands for antioxidative protection from ROS. Topcuoglu et al (38) demonstrated an elevation of plasma and tissue oxidative stress markers as a result of ovariectomy. In addition, hormone replacement therapies decreased oxidative stress markers in plasma and tissue of the OVX rats, suggesting a protecting effect of estrogens within the antioxidant defense systems (36). Contrary to the above mentioned studies, our results showed no impact of ovariectomy itself on the antioxidative and oxidative status in the rat liver. There were no significant differences in TBARS and GSH levels, as well as in the activity of antioxidative enzymes between OVX and SH rats. Other studies found contradictory results regarding the impact of ovariectomy on LPO and antioxidative enzyme activities (39,40). These differences may be ascribed to the use of different tissues, different ages of animals, and different times of ovariectomy, since Kankofer et al (14) showed dynamic changes in oxidative and antioxidative parameters during early development of estrogen insufficiency.

In our study, the response of hepatic oxidative stress markers to HFD was in accordance with that reported by Noeman et al (41), who showed significant increase in LPO and protein carbonyl levels, as well as a decrease in GSH levels and activity of GST and GPx enzymes in the liver of rats with HFD-induced obesity. In other reports, long-term feeding of a high-saturated fat diet induced oxidative stress, since it significantly attenuated the hepatic enzyme antioxidant system and increased the levels of LPO products in the liver (42). As shown in our study and the above mentioned studies (41,42), HFD causes a significant increase in biochemical indicators of liver damage, such as LPO. This could probably contribute to the additional progression of obesity-related problems (18,41). Feeding a HFD for long periods of time results in the occurrence of NAFLD, and hepatic lipid accumulation and oxidative stress are key pathophysiological mechanisms in this disease (18).

### Impact of PA and SOB on oxidative/antioxidative status in the liver of OVX rats fed with HFD

In our study, a special goal was also to examine the possible ameliorating effect of lifestyle modifications, such as PA and functional food containing bioactive compounds with enhanced antioxidative properties, on oxidative damage in the rat liver. Oxidative stress represents a potential attractive target for prevention and therapy of obesity-induced diseases. The training program used in this study did not attenuate oxidative damage caused by HFD in OVX and SH rats. PA did not induce any significant changes in TBARS and GSH levels, as well as in the activities of GPX, CAT and GST in the liver of SH and OVX animals. Although prolonged exercise may be protective due to activation and enhanced synthesis of antioxidants and antioxidant enzymes (43-45), results similar to ours were obtained by Rodrigues et al (46). Resistance training program used in their study did not attenuate the liver oxidative damage caused by ovariectomy and increased the hepatic oxidative stress (46).

Previous studies have described many health-beneficial effects of each bioactive compound (selenium in organic form, quercetin, curcumin, catechins) present in cereal SOB (47). Selenium up-regulates the major component of the antioxidant defense mechanism by controlling the GSH pool and some antioxidative enzymes (48). Antioxidant polyphenols (quercetin and catechins) can increase the antioxidant capacity of the body against obesity-induced oxidative stress directly, through scavenging ROS and chelating redox-active transition metal ions, and indirectly through inhibition of prooxidant enzymes and induction of antioxidant enzymes (49). In our study, SOB did not reduce negative impact of HFD on LPO and GSH levels, as well as on most of the antioxidative enzymes in the rat liver. SOB showed protective effect through increased GR activity in SH rats, and in OVX rats through increased GR activity. Also, SOB showed a tendency to increase GSH levels in rats. This impact of SOB on GSH levels and CAT activity could be attributed to selenium. The role of GR in reduction of oxidized glutathione back to the GSH is to maintain the level of intracellular GSH. Therefore, GR indirectly participates in protection of the cells against oxidative stress. It is also known that GR activity could be stimulated by the estrogens (50). Accordingly, it seems that estrogens together with bioactive compounds from SOB (selenium) were responsible for the increased GR activity in SH rats. In OVX rats, SOB increased CAT activity, while in SH rats CAT activity was also increased, but not significantly. The impact of SOB on increased CAT activity could be ascribed to quercetin and catechins. Flavonoids can bind to the heme moiety or a protein region of CAT and thus contribute to the enhancement of CAT activity (51). Madaric et al (28) found a beneficial effect of the same biscuits on cardiovascular risk markers in healthy population. The reduction of total cholesterol, LDL-cholesterol, atherogenic index, homocysteine, and asymmetric dimethylarginine was found after two months of biscuit consumption. Further studies should be performed in order to determine the possible therapies aimed at reducing oxidative damage induced by HFD and OVX.

No interaction effect on oxidative/antioxidative status was observed between HFD and ovariectomy. Feeding HFD was accompanied by decreased antioxidative enzymes activities and increased LPO in both OVX and SH rats. Decreased antioxidant defense suggests lowered oxidative stress resistance, which could be reflected in oxidative damage of the rat liver and metabolic disorders. Changes detected in the liver may reflect antioxidative/oxidative status of the whole body and the blood. Bioactive compounds of SOB showed a potential to attenuate the adverse impact of HFD by increasing activities of some antioxidative enzymes.
